# Potentially paraneoplastic glomerulopathies in a Brazilian cohort: a retrospective analysis

**DOI:** 10.1590/2175-8239-JBN-2024-0131en

**Published:** 2025-01-27

**Authors:** Marcella Soares Laferreira, Gianna Mastroianni Kirsztajn

**Affiliations:** 1 Universidade Federal de São Paulo (UNIFESP), Departamento de Medicina, Divisão de Nefrologia, São Paulo, SP, Brazil.

**Keywords:** Glomerulonephritis, Neoplasms, Kidney Neoplasms, Paraneoplastic Syndromes, Glomerulopathies, Glomerular Diseases, Membranous Glomerulopathy, Glomerulonefrite, Neoplasias, Neoplasias Renais, Síndromes Paraneoplásicas, Glomerulopatias, Doenças Glomerulares, Glomerulopatia Membranosa

## Abstract

**Introduction:**

Glomerular diseases can be associated with solid or hematopoietic malignancies. The prevalence of these associations varies according to the studied glomerular disease. This study aimed to evaluate the frequency and type of neoplasms in patients with glomerular diseases as well as their clinical, laboratory, and histopathological features and the relationship with immunosuppressive therapy.

**Methods:**

This was a retrospective, descriptive, observational, longitudinal study that reviewed 4,820 medical records and included 95 patients with glomerular disease and neoplasms. Demographic, clinical, laboratory, and histologic data were collected.

**Results:**

The prevalence of neoplasms was 1.97% (95 patients; 81 [85.3%] malignant, 14 [14.7%] benign). Hematologic malignancies (35.8%) showed the highest prevalence, followed by colon, rectal, and gynecologic tumors. The glomerulopathy with the highest frequency was membranous glomerulopathy (MGN, 25 patients, 35.7%). The dose of the immunosuppressive agents among patients with neoplasms before or after immunosuppression was not statistically different. Neoplasm was diagnosed before glomerulopathy in 53% of patients. Among cases in which neoplasms were diagnosed after glomerulopathy, 43% were diagnosed in the first year of follow-up of the renal disease. The predominant syndrome at presentation was nephrotic syndrome. Progression to chronic kidney disease stage 5 at the end of follow-up occurred in 8.4% of the cases.

**Conclusions:**

Neoplasms manifested before or, less frequently, after the diagnosis of glomerular diseases. As neoplasms diagnosed after presentation of glomerulopathy often appeared early after this diagnosis, it is necessary to be aware of neoplasms during the first year of follow-up of glomerulopathies, especially in patients with nephrotic syndrome, and MGN.

## Resumo

**Introdução:**

Doenças glomerulares podem estar associadas a malignidades sólidas ou hematopoiéticas. A prevalência dessas associações varia conforme a doença glomerular estudada. O objetivo deste estudo foi avaliar frequência e tipo de neoplasias em pacientes com doenças glomerulares, bem como suas características clínicas, laboratoriais e histopatológicas e a relação com a terapia imunossupressora.

**Métodos:**

Estudo retrospectivo, descritivo, observacional e longitudinal que analisou 4.820 prontuários e incluiu 95 pacientes com doença glomerular e neoplasias. Foram coletados dados demográficos, clínicos, laboratoriais e histológicos.

**Resultados:**

A prevalência de neoplasias foi 1,97% (95 pacientes; 81 [85,3%] malignas, 14 [14,7%] benignas). As malignidades hematológicas (35,8%) apresentaram maior prevalência, seguidas por tumores de cólon, reto e ginecológicos. A glomerulopatia com maior frequência foi a glomerulopatia membranosa (GM, 25 pacientes, 35,7%). A dose dos agentes imunossupressores entre pacientes com neoplasias, antes ou após a imunossupressão, não foi estatisticamente diferente. A neoplasia foi diagnosticada antes da glomerulopatia em 53% dos pacientes. Entre os casos em que as neoplasias foram diagnosticadas após a glomerulopatia, 43% foram detectadas no primeiro ano de acompanhamento da doença renal. A síndrome predominante na apresentação foi a síndrome nefrótica. A progressão para doença renal crônica estágio 5 ao final do acompanhamento ocorreu em 8,4% dos casos.

**Conclusões:**

Neoplasias se manifestaram antes ou, menos frequentemente, após o diagnóstico de doenças glomerulares. Como as neoplasias diagnosticadas após a apresentação da glomerulopatia frequentemente surgem logo após esse diagnóstico, é necessário atenção às neoplasias durante o primeiro ano de acompanhamento das glomerulopatias, especialmente em pacientes com síndrome nefrótica e GM.

## Introduction

Onconephrology is a new discipline that covers the interrelation between neoplasms and kidney diseases^1^. Glomerular diseases may be associated with solid or hematopoietic neoplasms^2^, and often represent the first clinical manifestation of an underlying cancer^3^.

The term “paraneoplastic syndrome” has been introduced to indicate clinical manifestations that are not directly related to tumor burden, invasion, or metastasis, but are caused by the secretion of tumor cell products such as hormones, growth factors, cytokines, and antigen tumor cells^4^. In 1922, Galoway^5^ introduced the concept of paraneoplastic glomerulopathy; however, the first original study highlighting the association between cancer and nephrotic syndrome was published in 1966 by Lee et al^6^. Since then, detailed reviews of glomerular diseases and neoplasms have been published^3,4,7,8,9,10,11^, but most reports on this association are based on small case series with a limited number of malign tumors, which does not allow adequate statistical analysis^12^.

The prevalence of neoplasms in patients with glomerulopathies varies from 5.2% to 14.1% in studies that encompassed the various glomerular diseases^13^. In a retrospective study, Heaf et al.^12^ analyzed 5594 patients combining the Danish Registry of Renal Biopsies with the National Oncology Registry. They investigated a total of 911 patients with neoplasms, among which 330 cases were diagnosed at kidney biopsy (36%). During the follow-up, 581 patients developed cancer (10.4%). The risk of cancer in these patients was about 3 times higher than that in the general population within a year before renal biopsy until 1 year after.

The relationships between glomerular diseases and neoplasms can follow different paths, as follows: neoplasms (with or without a previous diagnosis) causing glomerulopathies; immunosuppressive agents for the treatment of glomerular diseases triggering neoplasms; chemotherapy for the treatment of malignancies causing glomerulopathies; and viral infections inducing both glomerular diseases and malignancies^7^.

There are well-established associations between membranous glomerulopathy (MGN) and solid tumors^7^, between minimal change disease and Hodgkin’s lymphoma and thymoma^14,15^, and between membranoproliferative glomerulonephritis and chronic lymphoid leukemia^16^. Such associations are recognized as classic paraneoplastic glomerulopathies, but other glomerular diseases are also related to malignancies^17^.

The use of glucocorticoids, alkylating agents, calcineurin inhibitors, azathioprine, and mycophenolate is frequent during the treatment of primary glomerulopathies. The prevalence of malignancies after immunosuppressive treatment for glomerulopathies is much less studied, and the role of a single immunosuppressive drug in increasing the risk of neoplasm development is still poorly debated. However, it is known that the risk is greater with the use of carcinogenic drugs, and with more intensive and prolonged immunosuppressive treatment^3^. It is also possible that the initiation of immunosuppressive therapy for glomerular disease may cause a rapid progression of a pre-existing subclinical neoplasm^13^.

In the context of the relationship between malignancies and glomerular diseases described here, our study aimed to evaluate the occurrence of malignancies and the clinical, laboratory, and histopathological features of glomerulopathies in Brazilian patients who were followed up at the Federal University of São Paulo for >30 years.

## Methods

This was an observational, descriptive, retrospective cohort study based on the analysis of 4,820 consecutive physical medical records of patients enrolled in the Division of Nephrology (Glomerular Diseases Clinic) of the Federal University of São Paulo - Escola Paulista de Medicina (UNIFESP-EPM), a tertiary and academic reference center for glomerulopathies in São Paulo, Brazil.

The patients included in the study had a diagnosis of malignant or benign neoplasm and glomerular disease confirmed by abnormal laboratory results and/or kidney biopsy. Neoplastic diseases developed before or after the beginning of outpatient follow-up at the Glomerular Diseases Clinic. Patients of both sexes age 16 years or older were included. The exclusion criteria were a lack of description of malignancies either from anatomopathological examination or in medical records, or insufficient information about neoplastic and/or glomerular diseases in the medical records.

We selected 95 patients who had a diagnosis of neoplasm before or during outpatient follow-up. We evaluated the frequency and type of neoplasms in patients with glomerular diseases, the clinical, laboratory, and histopathological characteristics of glomerulopathies in patients diagnosed with neoplasms associated with glomerular diseases, and the relationship of immunosuppressive therapy for glomerular diseases and the occurrence of neoplasms.

The descriptive statistical analysis was initially done using median (range: minimum and maximum values), and absolute and relative frequencies (percentage). Inferential analyses were used to confirm or refute the evidence found in the descriptive analysis. The Mann-Whitney test was used for comparison of cumulative doses of immunosuppressive agents, according to the occurrence of neoplasm before and after the use immunosuppressive agent. Pearson’s chi-square test was used in the study of the association between oncologic cure and remission of glomerular disease. In all the inferential analyses, the alpha level of significance was 5%. Statistical analyses were carried out with the statistical program R version 3.5.1. This study was approved by the Ethics Committee of the Federal University of São Paulo. The study was planned and conducted in full compliance with the concepts of research ethics involving humans, including those mentioned in the Declaration of Helsinki.

## Results

From the 4,820 medical records reviewed, we initially found 102 patients with a diagnosis of neoplasm, both before the beginning of follow-up at our service and with onset during the follow-up of the glomerulopathy. Seven patients were excluded due to insufficient data, and 95 (1.97%) patients were evaluated. The sample predominantly consisted of female (55.8%) and white (61.1%) patients, with a median age of 55.0 years (range, 16.4–82.1 years). Most of these patients had hypertension (66.3%) and dyslipidemia (52.1%), with a median glomerular filtration rate (calculated using the Chronic Kidney Disease Epidemiology Collaboration equation) of 58.6 mL⋅min^−1^⋅1.73 m^−2^ (minimum 5.6, maximum 135.0). Table 1 summarizes some features of the studied population.

**Table 1:** General clinical and laboratory features of the studied population at admission (N = 95)

Gender, male	44.2%
Age, years*	55.0 (16.4–82.1)
Hypertension	66.3%
Dyslipidemia	52.1%
Diabetes mellitus	10.5%
GFR–CKD-EPI (mL⋅min^−1^⋅1.73 m^−2^)*	63.27 (5.6–135.0)
Hemoglobin (g/dL)*	12.40 (8–18.2)
24-h proteinuria (g)*	2.97 (0–21.0)

Abbreviation – GFR: glomerular filtration rate. Note – *median (range).

Fifty-one patients had the diagnosis of neoplasm before the first visit at the Glomerular Diseases Clinic was 51 (53.7%), and the median time between the neoplasm diagnosis and the initial consultation at our outpatient clinic was 24 months (range: 0–180 months). Of the 44 (46.3%) patients who had the diagnosis of neoplasm after the first visit, the mean time between the diagnosis of glomerular disease and that of neoplasm was 34.5 months (range: 1–219 months).

The most common sites of neoplasms in patients who presented with a neoplasm before admission were blood and blood forming tissue (45.1%), gynecologic organs (13.7%), colorectal region (7.8%), prostate (7.8%), breast (5.9%), and kidneys (5.9%). Of the 51 patients with neoplasm before glomerulopathy, 44 (86.3%) had undergone treatment, including surgery (61.4%), chemotherapy (59.1%), radiotherapy (18.2%), and hormone therapy (6.8%), with some patients having undergone more than one treatment. In patients diagnosed with neoplasm after the first visit, the time between glomerular disease and neoplasm diagnoses occurred in the first year in 43.2%, between the first and fifth year in 27.3%, and after the fifth year of the follow-up in 29.5%. Hematological neoplasms occurred in 25% of patients diagnosed after the start of follow-up in our group of glomerulopathies, and the other most common sites were: colorectal (11.4%), prostate (11.4%), thyroid (11.4%), breast (9.1%), and kidneys (9.1%).

Table 2 shows some information about neoplasms in patients with glomerulopathies. Of the 95 cases of neoplasms, 81 (85.3%) were malignant and 14 (14.7%) were benign. Oncologic cure was obtained in 64.9% of the patients. The neoplasms had several anatomopathological diagnoses, with a higher prevalence of multiple myeloma (10.5%), followed by non-Hodgkin’s lymphoma (9.5%), and prostatic adenocarcinoma (9.5%).

**Table 2:** Main information about the neoplasms in patients with glomerulopathies (GN)

Benign vs. malignant status (n = 95)	Benign	14	14.7%
	Malignant	81	85.3%
Timing of neoplasm diagnosis relative to GN (n = 95)	Before GN	51	53.7%
	After GN	44	46.3%
Oncologic cure (n = 94)	Yes	61	64.9%
Neoplasm diagnosis after immunosuppressive agent use (n = 44)	Yes	20	45.4%
Interval between immunosuppressive agent use and Neoplasm diagnosis (months)* (n = 20)		51.0 (2–161)	

Note – *Median (range).

With respect to the diagnosis of glomerulopathy, the most prevalent syndromes were nephrotic syndrome (28.4%), followed by non-nephrotic proteinuria (18.9%), renal function loss with nephrotic syndrome (16.8%), glomerular hematuria (15.8%), renal function loss with non-nephrotic proteinuria (11.6%), and nephritic syndrome (8.4%).

As summarized in Table 3, kidney biopsy was performed in 70 patients. Of these, it was possible to establish the histologic diagnosis of glomerular disease in 66 patients. The diagnosis with the highest frequency was MGN (25 patients, 35.7%). Focal segmental glomerulosclerosis (FSGS) and amyloidosis were detected in 12.9% and 11.4% of the renal biopsy specimens, respectively.

**Table 3:** Histological diagnoses based on kidney biopsies in patients with neoplasms

Histological diagnosis (n = 70)	MGN	25	35.7%
	FSGS	9	12.9%
	Amyloidosis	8	11.4%
	IgAN	5	7.1%
	MCD	5	7.1%
	MPGN	4	5.7%
	Advanced chronic nephropathy	3	4.3%
	Lupus Nephritis	3	4.3%
	Proliferative endocapillary GN	2	2.9%
	Crescentic pauci-immune GN	1	1.4%
	Thin basement membrane disease	1	1.4%
	Non representative material	4	5.7%

Abbreviations – MGN: membranous glomerulopathy; FSGS: focal segmental glomerulosclerosis; IgAN: IgA nephropathy; MCD: minimal change disease; MPGN: membranoproliferative glomerulonephritis; GN: glomerulonephritis.

Table 4 shows the distribution of associations of glomerular histologic diagnoses with sites and anatomopathological diagnoses of neoplasms.

**Table 4:** Summary of the associations of glomerular histological diagnoses with the sites and anatomopathological diagnoses of the neoplasms

Site of neoplasm	FSGS	MGN	IgAN	MCD	MPGN	Proliferative endocapillary GN	Amyloidosis	Advanced chronicity nephropathy	Crescentic GN	Thin membrane disease	Lupus nephritis	Non-representative material	Total
Colorectal region	–	6	1	–	–	–	–	–	–	–	–	–	7
Stomach	1	–	–	–	–	–	–	–	–	–	–	–	1
Liver	–	–	1	–	–	–	–	–	–	–	–	–	1
Gynecologic organs	1	2	1	1	1	–	–	–	–	–	–	–	6
Blood	2	3	–	2	1	2	7	2	1	–	1	–	21
Pituitary gland	–	–	–	–	1	–	–	–	–	–	–	–	1
Small intestine	–	2	–	–	–	–	–	–	–	–	–	–	2
Breast	1	1	–	1	–	–	–	1	–	–	–	2	6
Oropharynx	–	2	–	–	–	–	–	–	–	–	–	–	2
Skin	–	–	–	–	–	–	–	–	–	–	1	2	3
Penis	1	–	–	–	–	–	–	–	–	–	–	0	1
Prostate	1	4	1	–	–	–	1	–	–	–	–	–	7
Lung	–	2	–	–	1	–	–	–	–	–	–	–	3
Kidney	1	–	1	1	–	–	–	–	–	–	1	–	4
Thyroid	1	3	–	–	–	–	–	–	–	1	–	–	5
Total	9	25	5	5	4	2	8	3	1	1	3	4	70
Colorectal adenocarcinoma	–	3	–	–	–	–	–	–	–	–	–	–	3
Prostatic adenocarcinoma	1	4	1	–	–	–	1	–	–	–	–	–	7
Colorectal adenoma	–	2	1	–	–	–	–	–	–	–	–	–	3
Small–bowel adenoma	–	1	–	–	–	–	–	–	–	–	–	–	1
Pituitary adenoma	–	–	–	–	1	–	–	–	–	–	–	–	1
Primary amyloidosis	–	–	–	–	–	–	4	–	–	–	–	–	4
Basal cell skin Ca	–	–	–	–	–	–	–	–	–	–	1	2	3
Uterine Ca	–	1	1	–	–	–	–	–	–	–	–	–	2
Breast Ca	–	1	–	–	–	–	–	–	–	–	–	2	3
Oropharyngeal Ca	–	2	–	–	–	–	–	–	–	–	–	–	2
Lung Ca	–	1	–	–	–	–	–	–	–	–	–	–	1
Thyroid Ca	1	2	–	–	–	–	–	–	–	1	–	–	4
Hepatic Ca	–	–	1	–	–	–	–	–	–	–	–	–	1
Renal Ca	1	–	1	–	–	–	–	–	–	–	1	–	3
GIST	1	–	–	–	–	–	–	–	–	–	–	–	1
Chronic myeloid leukemia	–	–	–	–	–	1	–	–	–	–	–	–	1
Hodgkin's lymphoma	–	–	–	1	–	–	–	–	–	–	–	–	1
Non–Hodgkin's lymphoma	–	1	–	–	1	1	–	1	1	–	1	–	6
MGUS	–	1	–	–	–	–	–	–	–	–	–	–	1
Multiple myeloma	–	1	–	1	–	–	3	1	–	–	–	–	6
Myelodysplastic syndrome	2	–	–	–	–	–	–	–	–	–	–	–	2
Intestinal neuroendocrine tumor	–	1	–	–	–	–	–	–	–	–	–	–	1
Without report	3	4	–	3	2	–	–	1	–	–	–	–	13
Total	9	25	5	5	4	2	8	3	1	1	3	4	70

Abbreviations – Ca: carcinoma; FSGS: focal segmental glomerulosclerosis; MGN: membranous glomerulopathy; IgAN: IgA nephropathy; MCD: minimal change disease; MPGN: membranoproliferative glomerulonephritis; GN: glomerulonephritis; GIST: gastrointestinal stromal tumor; MGUS: monoclonal gammopathy of undetermined significance

The anti-PLA2r antibody was investigated in some patients with MGN (48%), and 9 (75%) of these patients were negative for anti-PLA2r antibodies.

Of the 95 patients analyzed, 64 (67.4%) used antiproteinuric drugs (angiotensin-converting enzyme inhibitors and/or angiotensin receptor blocker). The use of immunosuppressive agents was recorded in 34 patients (35.8%), of whom 32 were treated with corticosteroids (intravenous and oral). Cyclophosphamide was used by 18 patients, with an average duration of use of 3 months and an average cumulative dose of 11.53 g. The use of other immunosuppressants was also reported, including cyclosporine, azathioprine, mycophenolate, and rituximab.

Twenty (21.1%) patients had the diagnosis of neoplasm after the use of some immunosuppressive drugs, with the median time between the use of the medication and the diagnosis of neoplasm of 51 months.

We compared the doses of each immunosuppressant among those who had the diagnosis of neoplasm before immunosuppression and those after immunosuppression using the Mann-Whitney test. No difference between groups was found (as shown in Table S1).

As summarized in Table 5, by the end of follow-up, approximately 54 (56.8%) patients had no remission of glomerulopathy, 20 (21.1%) had partial remission, and 21 (22.1%) had total remission. Among remission cases, 32 (78.0%) were induced remissions and 9 (22.0%) were spontaneous remissions, and only 5 (12.2%) had recurrence.

**Table 5:** Renal outcomes of patients with glomerulopathies and neoplasms

Total follow-up time (months)* (n = 94)	41.58 (0.99–359.00)		
Presence of remission (n = 95)	Without remission	54	56.8%
	With partial remission	20	21.1%
	With total remission	21	22.1%
Type of remission (n = 41)	Induced	32	78.0%
	Spontaneous	9	22.0%
Remission of GN according to oncologic cure (n = 94)	Without remission (n = 53)	34	64.2%
	With partial remission (n = 20)	12	60.0%
	With total remission (n = 21)	15	71.4%
Recurrence (n = 41)	Yes	5	12.2%
End-stage kidney disease (n = 95)	Yes	8	8.4%
Doubling of serum creatinine level (n = 95)	Yes	9	9.5%

Abbreviation – GN: glomerulopathies. Note – *Median (range).

Oncologic cure was not associated with the remission of glomerular disease (without vs. partial or total remission, p = 0.735).

## Discussion

In our study, we found a 1.97% prevalence of neoplasms associated with glomerulopathies. This rate varies from 5.2% to 14.1% in studies that analyzed the various glomerular diseases combined^12,13^, and from 4% to 21%^17,18,19,20,21,22,23,24,25^ when it is restricted to patients with MGN. In the largest study on MGN and malignant neoplasms, Lefaucheur et al.^18^ demonstrated a 10% prevalence of malignancies in 240 patients with MGN. Of these, only half had symptoms related to the neoplasm at the time of renal biopsy. In 2014, Leeaphorn et al.^25^ performed a meta-analysis of studies on patients with MGN and malignancies in 785 patients. The prevalence of malignancies was 10%. It was also confirmed that the prevalence of solid malignancies was 86% against 14% prevalence of hematologic malignancies among patients with MGN.

In fact, the prevalence of neoplasms associated with glomerulopathies varies widely among the various studies, as it depends on the age of the studied population, the type of glomerular lesion analyzed, and the methodology used.

The diagnosis of neoplasms preceded glomerulopathy diagnosis in 53% of the cases; in the others, neoplasms were diagnosed after the diagnosis of glomerulopathy. Malignancies predominated in the medical records of these patients with glomerulopathies. Some benign neoplasms, such as polycythemia vera and essential thrombocytosis, were identified and excluded from this study. It is worth mentioning that there is a possibility of bias, because it is easier for patients to report malignant than benign neoplasms, and both patients and physicians might not place sufficient importance on registering benign lesions in medical records. In fact, most of the published studies on this topic excluded patients with benign neoplasms. Therefore, we could not find adequate data to make comparisons. In the patients that already had neoplasm diagnosis before the first visit in the nephrology service, hematologic malignancies predominated (45%), followed by gynecologic, colorectal, and prostatic tumors. Most of the neoplasms (86%) had been previously treated, mainly with surgery and chemotherapy. In those who developed a neoplasm during follow-up in the nephrology service, a higher prevalence of hematologic malignancies (25%) was also observed, followed by colon, prostate, and thyroid neoplasms. Almost all of these patients received some type of treatment (97.8%). The predominance of hematologic malignancies, such as multiple myeloma and amyloidosis, may be due to the fact that kidney biopsy itself allows such oncologic diagnoses^12^. Hematologic tumors were not frequent among patients with MGN, in whom solid tumors were predominant, such as colon, rectal, prostate, and thyroid tumors.

It is interesting to note that, among the neoplasms that arose during the follow-up in the nephrology service, 43.2% were diagnosed within one year of the glomerulopathy diagnosis, similar to what was reported by Heaf et al.^12^ This percentage suggests that, in these cases, the neoplasm was coexisting with the glomerular disease and was not due to the use of immunosuppressive drugs.

Of the 95 patients with glomerular disease and neoplasms, only 70 underwent a renal biopsy. The glomerulopathy that was most associated with malignancies was MGN. As this is a well-established relationship^7,8,12^, there is usually a greater demand for neoplasm screening in these cases, which may eventually contribute to the increase in the diagnoses of asymptomatic malignancies. Another bias related to the higher frequency in MGN would be the fact that both glomerular disease and oncologic disease are more frequent in adults after the fifth or sixth decade of life. There is no consensus about screening for malignancies in this population, and it should be conducted according to age, sex, and inherent risk factors of each patient. In this study, it was not possible to obtain information on all the screening tests applied in this MGN population.

Advances in the understanding of MGN pathogenesis and availability of biomarkers to differentiate primary from secondary MGN are an important contribution to the current investigation of this glomerular disease^11,26,27,28,29^. The anti-phospholipase A2 receptor (anti-PLA2r) antibody is present in 75–80% of cases of primary MGN^27^. New biomarkers for MGN are being discovered, including some with stronger associations with malignancies, such as anti-thrombospondin type 1 domain-containing 7A (anti-THSD7A) antibody, which was detected in approximately 5–12% of patients with MGN who are anti-PLA2r negative^11,27^. A systematic review, involving 4,121 patients with MGN, found an incidence of malignancy between 6–25% in anti-THSD7A positive patients^30^. Another potential new biomarker in the diagnosis of MGN-related malignancy is neural epidermal growth factor-like 1 protein (NELL-1) mainly in older patients^31,32^.

In some patients with MGN, it was possible to test for the anti-PLA2r antibody, and the results were negative in 75% of the cases. It should be emphasized that the absence of this antibody is expected in secondary MGN, including MGN secondary to malignancies.

MGN was associated mostly with colon-rectal carcinoma and prostate adenocarcinoma. Regarding other glomerular diseases diagnosed in kidney biopsy, we found a great variation in the localization of the neoplasms. As there were few patients with FSGS, minimal change disease, and membranoproliferative glomerulonephritis, it was not possible to establish any causal associations with these other histologic types.

Regarding treatment, more than 37% of the patients in the present study used antiproteinuric medications. The use of immunosuppressants was recorded in 36% of the patients. This percentage was probably not higher, even in a large part of our patients presenting with nephrotic syndrome, because more than half of them had a previous diagnosis of malignancy. In this scenario, we tend to be more conservative and, in general, immunosuppressive therapy is not indicated.

Only 21% of the neoplasms diagnosed in this study occurred after immunosuppression, between 2 and 161 months after the initiation of such treatment. With this wide variation in the timing of diagnosis and considering the diversity of diseases, it is difficult to attribute the development of a particular neoplasm to the use of certain immunosuppressive medications. After immunosuppression, hematologic malignancies predominated, followed by breast and prostate carcinomas.

It is known that the use of corticosteroids, which are not considered oncogenic drugs, can suppress cellular immunity and also partially inhibit humoral immunity. Cyclophosphamide is an alkylating agent and its use is associated with bladder cancer and hematological malignancies. Such oncogenic effect is considered dose-dependent^12^, which is why pre-defined maximum doses should not be exceeded in the treatment of glomerular diseases. Azathioprine and calcineurin inhibitors are considered possible causes of neoplasia in organ recipients^3^. On the other hand, the use of mycophenolate has been associated with a reduction in the incidence of lymphoproliferative disorders^33^.

The comparison of the cumulative doses of each immunosuppressant showed no statistical difference between patients who developed a neoplasm before and after immunosuppression. However, we cannot rule out the possibility that immunosuppressive therapy can speed up the progression from a subclinical lesion to a malignant neoplasm. The relatively small number of patients with neoplasms in our sample may have interfered with this outcome. There are no robust studies defining the risk of neoplasm development in a population that has used immunosuppressants for the treatment of glomerular diseases. Many data refer to the use of immunosuppressants for patients undergoing renal transplantation and/or for other diseases^3^.

The median follow-up time of the patients was 41.6 months, and the outcomes were evaluated at the time of the last visit recorded in the medical chart. With respect to renal outcomes, total or partial remission of glomerular disease was achieved in 43% of our patients. It is described that in some cases, proteinuria may persist despite tumor removal, which may be due to established structural changes in the kidneys^25^.

Among the cases of neoplasm-associated glomerulopathies, 8.4% progressed to end-stage kidney disease at the end of follow-up. We did not consider death as an outcome, as our study was based on data from outpatient records, where such information is not commonly present.

There was no clear association between oncologic cure and remission of glomerulopathy in the total sample, even when analyzing only patients with MGN. In fact, although neoplasm-associated glomerulopathy is expected to improve after specific treatment, many cases did not show remission of glomerular disease, with both conditions evolving independently^34^.

We recognize that our study had limitations. These include the inclusion of patients with and without renal biopsy, as information on both situations would be relevant, since patients without biopsy had a well-established clinical and laboratory diagnosis of glomerulopathy. These corresponded to syndromic diagnoses of non-nephrotic proteinuria, proteinuria associated with renal function deficit, and/or glomerular hematuria. As this was a retrospective analysis, which in itself is another limitation, the reasons for not having undergone renal biopsy varied, including the positioning of the medical team in relation to biopsy indications throughout the study period. Also, due to the retrospective nature of this study, more recent markers such as anti-PLA2R autoantibodies were not available in more cases. At last, the diversity of diagnoses of neoplasms and glomerular diseases restricted the detection of associations between the two groups of diseases. On the other hand, the number of patients screened and included is certainly a strength of our study.

Considering that neoplasms were detected early after the diagnosis of glomerular disease in this study, we emphasize that it is necessary to be aware of such diagnosis during the first year of glomerulopathy follow-up, particularly in patients with nephrotic syndrome and especially with conditions caused by MGN in patients >50 years of age.

## Conclusions

Neoplasms correlate with glomerulopathies in several ways and may manifest before or after the diagnosis of glomerular diseases. Several histological diagnoses of glomerulopathies are associated with malignancies, but the most prevalent is MGN. Although clinical, laboratory, and histological features may help differentiate primary from paraneoplastic glomerulopathies, biomarkers are helpful and there are new and promising tests in this area.

## Supplementary Material

The following online material is available for this article:

Table S1 - Comparison of the cumulative doses of each immunosuppressant between those who had neoplasm before and after immunosuppression.
