# Supplementary material for: Potentially paraneoplastic glomerulopathies in a Brazilian cohort: a retrospective analysis
Source: J Bras Nefrol. 2025 Jan 27;47(1):e20240131. doi: 10.1590/2175-8239-JBN-2024-0131en (PMC11781679; doi:10.1590/2175-8239-JBN-2024-0131en)
Supplement: Supplementary file 1 [file 2175-8239-jbn-47-1-e20240131-suppl1.pdf]

Braz. J. Nephrol.
https://doi.org/10.1590/2175-8239-JBN-2024-0131pt

Material Suplementar para "Potenciais glomerulopatias paraneoplásicas em uma coorte brasileira: uma análise retrospectiva"

Tabela S1 - Comparação das doses cumulativas de cada imunossupressor entre aqueles que apresentaram neoplasia antes e após a imunossupressão.

Dose cumulativa (g)		antes da ISS	após a ISS	p
Corticosteroide	n	12	20	0,716[a]
	mediana	3,16	3,11	
	mínimo	0,03	0,06	
	máximo	12,49	13,27	
Ciclofosfamida	n	3	15	0,824[a]
	mediana	9,20	11,50	
	mínimo	9,10	1,09	
	máximo	13,80	27,45	
Ciclosporina	n	-	8	-
	mediana	-	206,90	
	mínimo	-	18,40	
	máximo	-	922,50	
Azatioprina	n	2	7	0,222[a]
	mediana	36,60	68,25	
	mínimo	18,30	21,20	
	máximo	54,90	355,95	
Micofenolato	n	2	4	>0,999[a]
	mediana	514,44	262,80	
	mínimo	65,52	65,88	
	máximo	963,36	525,60	
Rituximabe	n	-	2	-
	mediana	-	1,00	
	mínimo	-	1,00	
	máximo	-	1,00	

[a]Teste de Mann-Whitney; ISS: imunossupressão.
