# Supplementary material for: Potentially paraneoplastic glomerulopathies in a Brazilian cohort: a retrospective analysis
Source: J Bras Nefrol. 2025 Jan 27;47(1):e20240131. doi: 10.1590/2175-8239-JBN-2024-0131en (PMC11781679; doi:10.1590/2175-8239-JBN-2024-0131en)
Supplement: Supplementary file 2 [file 2175-8239-jbn-47-1-e20240131-suppl2.pdf]

*Braz. J. Nephrol.*
https://doi.org/10.1590/2175-8239-JBN-2024-0131en

# Supplementary Material to "Potential paraneoplastic glomerulopathies in a Brazilian cohort: a retrospective analysis"

**Table S1** - Comparison of the cumulative doses of each immunosuppressant between those who had neoplasm before and after immunosuppression.

| Cumulative dose (g) | | before ISS | after ISS | p |
|---|---|---|---|---|
| Corticosteroid | n | 12 | 20 | 0.716[a] |
| | median | 3.16 | 3.11 | |
| | minimum | 0.03 | 0.06 | |
| | maximum | 12.49 | 13.27 | |
| Cyclophosphamide | n | 3 | 15 | 0.824[a] |
| | median | 9.20 | 11.50 | |
| | minimum | 9.10 | 1.09 | |
| | maximum | 13.80 | 27.45 | |
| Cyclosporine | n | - | 8 | - |
| | median | - | 206.90 | |
| | minimum | - | 18.40 | |
| | maximum | - | 922.50 | |
| Azathioprine | n | 2 | 7 | 0.222[a] |
| | median | 36.60 | 68.25 | |
| | minimum | 18.30 | 21.20 | |
| | maximum | 54.90 | 355.95 | |
| Mycophenolate | n | 2 | 4 | >0.999[a] |
| | median | 514.44 | 262.80 | |
| | minimum | 65.52 | 65.88 | |
| | maximum | 963.36 | 525.60 | |
| Rituximab | n | - | 2 | - |
| | median | - | 1.00 | |
| | minimum | - | 1.00 | |
| | maximum | - | 1.00 | |

[a]Mann-Whitney test; ISS: immunosuppression.
